# Bio-inspired antimicrobial surfaces fabricated by glancing angle deposition

**DOI:** 10.1038/s41598-022-27225-4

**Published:** 2023-01-05

**Authors:** Chuang Qu, Jesse L. Rozsa, Hyun-Jin Jung, Anna R. Williams, Emmanuel K. Markin, Mark P. Running, Shamus McNamara, Kevin M. Walsh

**Affiliations:** 1 Department of Electrical and Computer Engineering, University of Louisville, 2210 S Brook St, Louisville, KY 40292 USA; 2 Department of Biology, University of Louisville, 139 Life Sciences Bldg., Louisville, KY 40292 USA

**Keywords:** Nanofabrication and nanopatterning, Nanostructures, Synthesis and processing, Nanostructures, Antimicrobials

## Abstract

This paper describes the fabrication of cicada-wing-inspired antimicrobial surfaces using Glancing Angle Deposition (GLAD). From the study of an annual cicada (*Neotibicen Canicularis*, also known as dog-day cicada) in North America, it is found that the cicada wing surfaces are composed of unique three-dimensional (3D) nanofeature arrays, which grant them extraordinary properties including antimicrobial (antifouling) and antireflective. However, the morphology of these 3D nanostructures imposes challenges in artificially synthesizing the structures by utilizing and scaling up the template area from nature. From the perspective of circumventing the difficulties of creating 3D nanofeature arrays with top-down nanofabrication techniques, this paper introduces a nanofabrication process that combines bottom-up steps: self-assembled nanospheres are used as the bases of the features, while sub-100 nm pillars are grown on top of the bases by GLAD. Scanning electron micrographs show the resemblance of the synthesized cicada wing mimicry samples to the actual cicada wings, both quantitatively and qualitatively. The synthetic mimicry samples are hydrophobic with a water contact angle of 125˚. Finally, the antimicrobial properties of the mimicries are validated by showing flat growth curves of *Escherichia coli* (*E. coli*) and by direct observation under scanning electron microscopy (SEM). The process is potentially suitable for large-area antimicrobial applications in food and biomedical industries.

## Introduction

In 2021, the emergence of Brood X cicada, which occurs every 17 years in the eastern United States, sparked new research interest on the insect. Cicada wings possess protective natural traits, including water repellent^1^, camouflage/antireflection^2,3^, and antimicrobial/antifouling^4^, that could also be beneficial to human beings. These behaviors are attributed to the natural intricate three dimensional nanostructures on the wings of cicadas. The bacteria-killing mechanism of the cicada wings has been introduced in previous publications^4–6^. In contrast to some natural/synthesized antimicrobial surfaces, such as metallic nanoparticles^7–9^, copper^10^, and diamond^11^, which are chemical based, the bacteria-killing mechanism of the cicada wings is solely physical and material independent. The nanostructure arrays on the wing surfaces are able to puncture the cellular membranes of the bacteria that land on top of them with the widths of around 1 μm, and eventually disintegrate the bacteria.

Cicada wings offer a good design template for synthetic antimicrobial surfaces. However, challenges emerge when attempting to artificially synthesize the same structures for practical applications. First of all, the features on the surfaces of cicada wings are mostly three-dimensional, sub-100 nm and densely packed. These requirements make it difficult to use conventional nanofabrication techniques such as top-down and bottom-up techniques. Conventional top-down techniques, such as electron beam lithography, are not suitable for creating three-dimensional nanostructures, and bottom-up techniques such as self-assembly lack the ability to do fine control of nanofeatures. Additionally, scaling-up of the area of nanofeature arrays could be time-consuming and cost-prohibitive. Research groups are combining both top-down and bottom-up techniques to create cicada wing nanostructures, by, for example, self-assembly and etching^12–14^. Nonetheless, the previously attempted processes could not completely recreate the nanostructure of the cicada wings. Another approach to achieve antimicrobial surfaces by mimicking cicada wings is to directly use cicada wings as the template and molding and remolding, such as described in previous publications^15–17^. However, this process still suffers from the problem of the limitation of scaling: the area of the antimicrobial surfaces is the same as the area of the wings.

In this paper, we propose to a novel two bottom-up process for recreating cicada wing structures on larger areas- Glancing angle deposition (GLAD) with self-assembled pre-determined seeds. GLAD is a unique physical vapor deposition (PVD) process that is capable of creating complicated three-dimensional nanofeatures such as columns, springs, chevrons, ribbons, combined features, and nanoporous structures^18,19^. GLAD is applicable to variable materials and large, flexible substrates. “Seeding” is an important concept in GLAD because it determines the distribution and density of the GLAD nanofeatures. When the substrate is flat prior to depositions, natural seeds will be formed and randomly distributed on the substrate, and the resulting grown GLAD features will also be randomly distributed. On the other hand, artificial nucleation sites can be prepared prior to depositions allowing for periodical GLAD features to be achieved on periodical artificial nucleation sites. Various types of seeds and seeding strategies are available for producing different GLAD features. The most commonly used seeds are flat-top cylinder seeds^20,21^, sphere seeds^22,23^, and line seeds^24,25^. According to the rules for GLAD seed design, the size of the GLAD features will follow the size of the flat-top cylinder seeds, which are at the 100 nm scale and require nanofabrication techniques for creating the seeds. When the top surfaces of the seeds are not flat, such as line seeds and sphere seeds, the size of the GLAD features grown on top do not necessarily follow the size of the seeds. This gives the opportunity for GLAD to create hierarchical three-dimensional nanostructures. Our group has proposed the seeding rules for sphere seeds for GLAD^23^ and discovered the size dependence of the GLAD features, which is suitable for recreating the nanopillar-sphere base structures as the cicada wing mimicry. The seeding of GLAD sphere seeds relies on self-assembly of nanospheres. Common processes of self-assembly of nanoparticles for example, spin-coating^26^, micro-propulsive injection^27^, and air–water interface^28^ are proposed in previous publications; self-assembled monolayer nanoparticle arrays over 1 m^2^ have been reported^27^.

In this paper, the template of the cicada wing for antimicrobial surfaces is introduced. With the template of antimicrobial surfaces determined, the whole design and fabrication process to obtain the mimicry of the cicada wing template by GLAD with sphere seeds are proposed. Initial characterizations of the synthetic mimicries, including the morphology of the synthetic mimicries and the water contact angles of the synthetic mimicry surfaces are conducted. Finally, the antimicrobial test is conducted using *E. coli*, and the antimicrobial property of the synthetic mimicry samples is shown.

## Methods

### Design and fabrication of sphere seeds for glancing angle deposition

**Figure 1:**
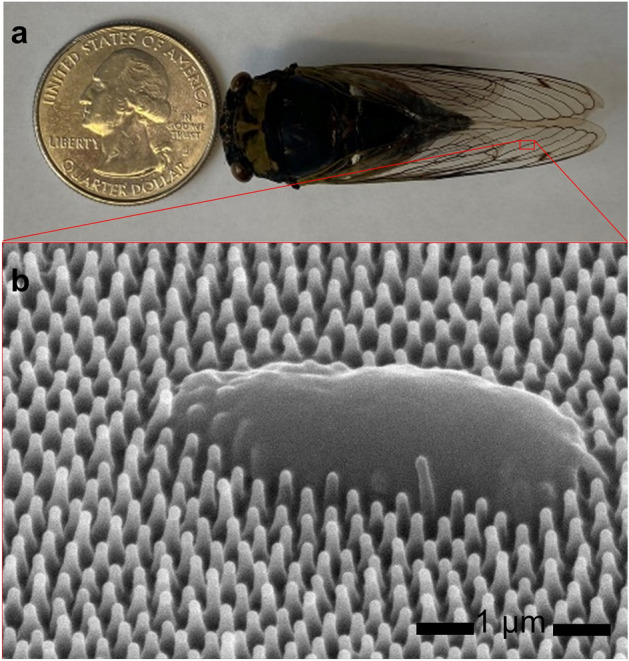
**(a)** Image of an annual cicada (*Neotibicen canicularis*) compared to a US quarter. (**b**) SEM image of the nanostructures on the wing surface of the cicada with *E. coli* on the wing surface.

The design of the antimicrobial surface is inspired by cicada wings. *Neotibicen Canicularis*, also known as the dog-day cicada (Fig. 1a), is an annual cicada species that appears in North America and is studied in our research. The area of the cicada wing is similar to that of a US quarter, as Fig. 1a indicates. As shown in the SEM image in Fig. 1b, the wing is composed of so called ‘nanopillar cones’ that are poly-crystalline distributed. The individual nanopillar cone has two parts: the spherical base, and the cylinders on top. The bases are about 180 nm apart from their neighbors, and the diameter of each is ~ 150 nm. The cylinders are about 90 nm in diameter and 200 nm in height. Figure 1b also shows the relative sizes of the nanopillar cones compared to *E. coli*. The width of the *E. coli* is around 900 nm; the bacteria are punctured by the needle-like nanopillar cones and eventually killed.

To replicate the three-dimensional nanostructures, the two parts of the nanopillars cones are created separately. Self-assembled nanospheres are used as the bottom bases, and sub-100 nm pillars can be grown from the bases by glancing angle deposition. However, the seeds for GLAD need to be designed properly to guarantee a uniform deposition over all the seeds, so seeding rules are required. Seeding rules specifically for GLAD three-dimensional seeds were previously developed by our research group, including sphere seeds^23^ and line seeds^25^ for GLAD. The factors that need to be taken into consideration when designing sphere seeds for GLAD are: inter-distances among neighboring seeds, diameter of the seeds, and the incident angle during GLAD. By proper shadowing calculations, the deposition area and diameter can be calculated, as can the percent coverage on the seeds, which determines the uniformity of deposition.

Since the nanospheres are hexagonally- and closely-packed after self-assembly, the distance between the centers of the neighboring seeds is the diameter of the nanospheres used. The distance can be easily changed by using different sized nanospheres. In the design to replicate cicada wing structures, 200 nm diameter nanospheres are chosen to match the inter-distances of the nanopillar cones. Once the distance is determined, the diameter of the nanospheres can be further reduced by isotropic gaseous etching to allow them to be separated from each other and reducing their size accordingly. The percent coverage can also be altered by changing the size of the nanosphere seeds in order to have more uniformly deposited pillars. Nanospheres with a finalized diameter of 156 nm are desired for the separated spherical bases as seeds for GLAD.

All the fabrication processes were conducted in a class-100 cleanroom. The nanospheres (Suzhou Nanomicro Technology) used were polystyrene, 200-nm in diameter (nominal diameter 194.5 nm with 1.81% CV), in aqueous solution (weight percentage of nanospheres is 1%). Surfactant of poly(ethylene glycol) (12) tridecyl ether (Sigma Aldrich) was firstly diluted in de-ionized water with a volumetric ratio of 1:400. The nanosphere aqueous solution was mixed with the surfactant solution with volumetric ratio of 1:3. The final mixture was then dispensed on a 1 inch × 1 inch glass slide. The nanospheres were self-assembled on the substrate by spin-coating with the following recipe: 150 rpm for 120 s, 250 rpm for 120 s, 800 rpm for 60 s, 2500 rpm for 60 s, and 5000 rpm for 10 s, with the acceleration of 200 rpm/s. The spinning speed was slow at first to spread the nanospheres, and then was gradually increased to 5000 rpm for drying the spheres.

The sizes of the nanosphere seeds were further reduced by oxygen plasma etching (Trion). The parameters on the etchers were: base pressure 50 mTorr, RIE power 50 W, ICP power 160 W, and the flow rate of O_2_ was 20 sccm. After 20 s of etching, an array of nanospheres with a diameter of 156 nm was obtained and ready for deposition.

### GLAD process

**Figure 2:**
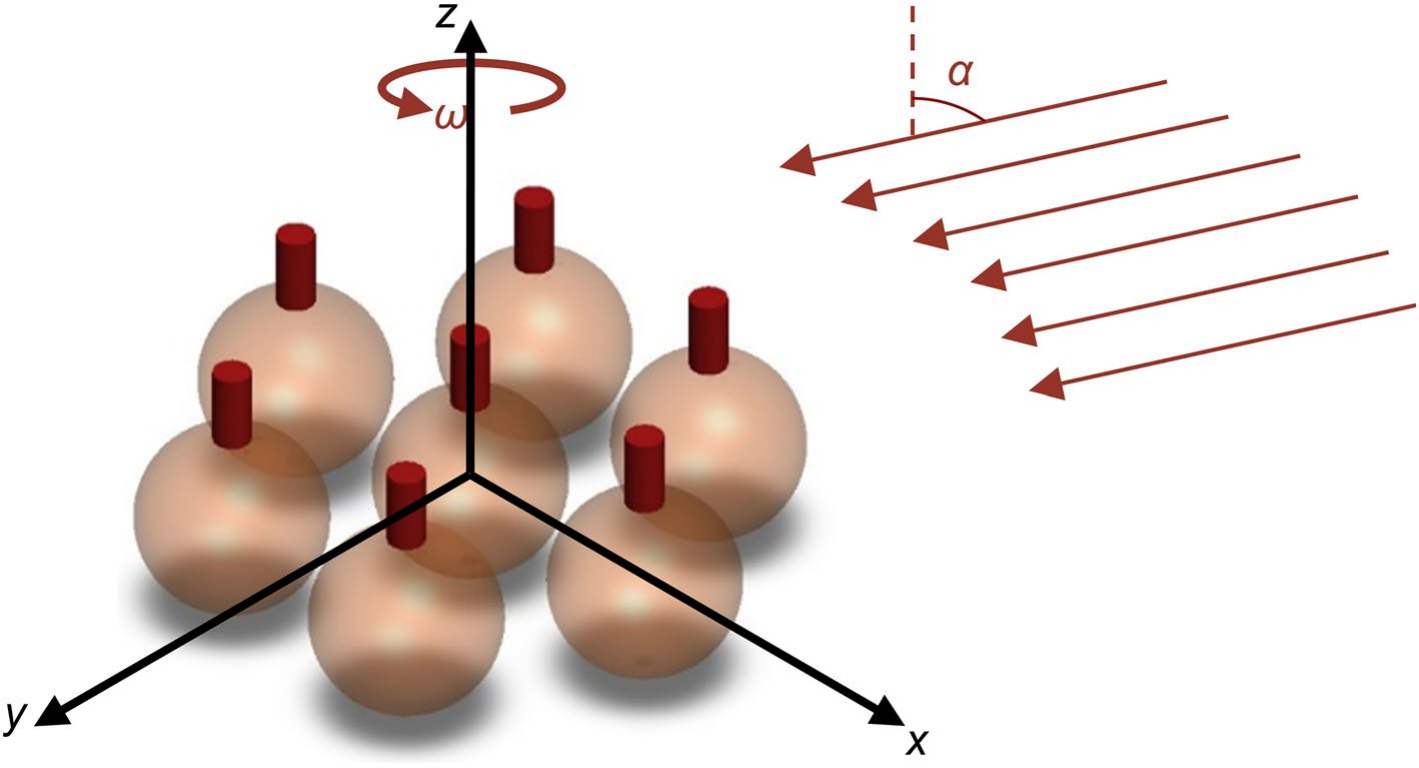
GLAD growth mechanism on sphere seeds. The vapor (arrows) comes with an incident angle of *α* and deposits on sphere seeds while the substrate rotates at the rate *ω*.

The growth mechanism of GLAD on sphere seeds is shown in Fig. 2. GLAD was conducted by electron beam evaporation (Kurt Lesker). The chamber pressure was pumped down to 10^–6^ Torr prior to depositions. The incident angle was determined as 85º in the seed design process for achieving sub-100 nm and uniform nanopillars on the sphere seeds^23^. The rotation speed was set to 5 rpm for creating cylindrical shapes. Helix-like features are available when the rotation is as slow as 0.04 rpm. The nanopillars were grown to around 200 nm to match the actual cicada wing features.

The material deposited for the samples was germanium. Since the bacterial killing mechanism is physical puncturing and rupturing, antimicrobial properties are not required for the pillar material. GLAD is suitable for a variety of materials including titanium^29^, silicon^30^ and silicon dioxide^31^, and germanium is used in this paper for demonstration purposes only.

To enhance adhesion between the nanopillars and the sphere bases, a protection layer was added to the samples. Parylene C monomer of 25 g was prepared for the coating of ~ 50 nm of a conformal layer on top of the nanostructures using a parylene coating system (Specialty Coating System).

### Characterization methods

**Figure 3:**
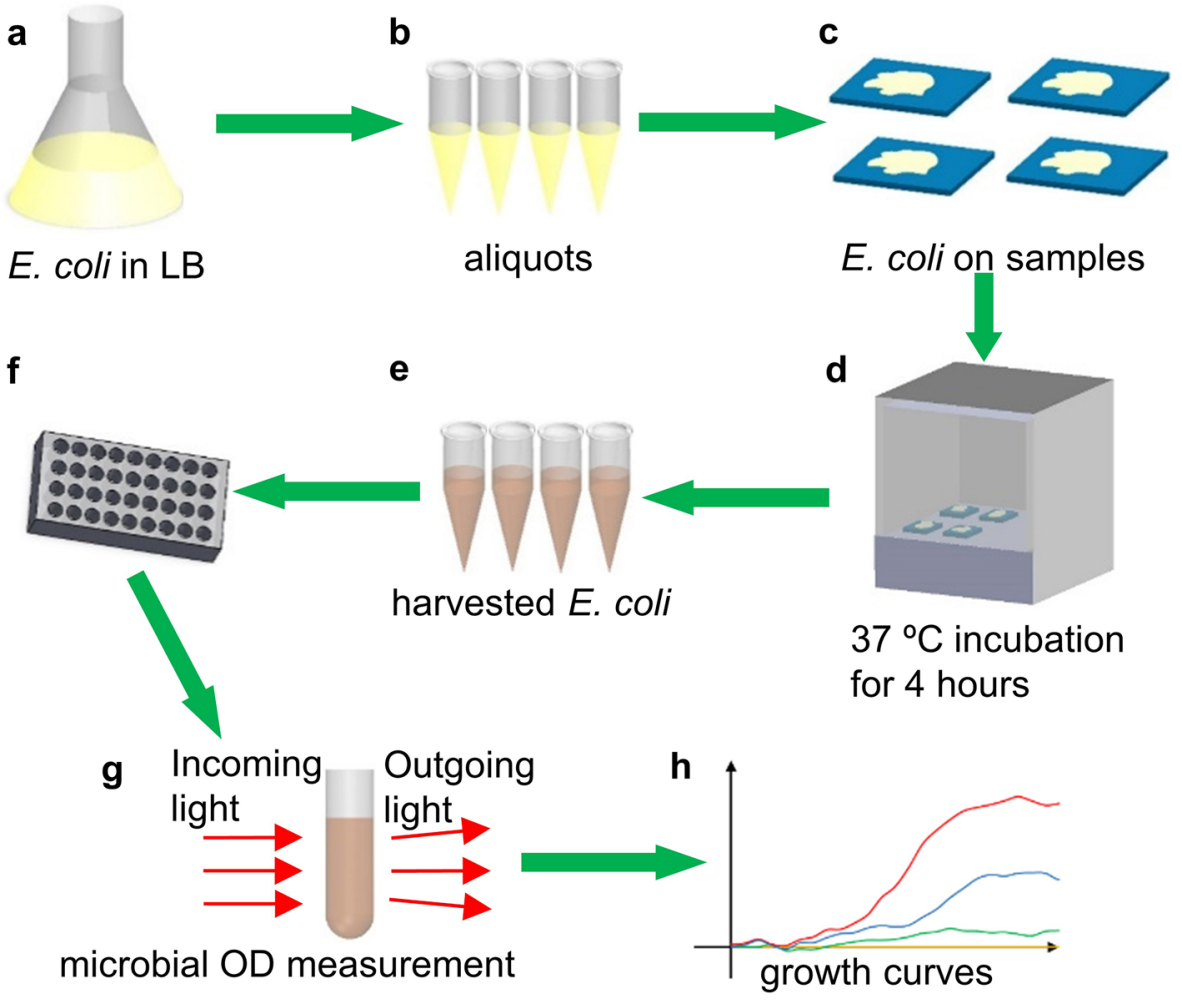
Procedures of the biological test. (**a**) Cultivating *E. coli*, (**b**) aliquots of *E. coli* after dilution, (**c**) application on samples, (**d**) incubation at 37° for 4 h, (**e**) dilution after harvesting *E. coli* from samples, (**f**) dispensing into test tubes, (**g**) microbial OD measurement, (**h**) growth curves obtained.

Figure 3 shows the procedure to perform the bacteria tests. The antibacterial performances of synthesized cicada-wing mimicry samples A-C, a negative control sample of a copper sheet (which is known for having antimicrobial properties), and a positive control sample of a glass slide (which is known for NOT having antimicrobial property) were analyzed. *Escherichia coli* (*E. coli*), a common Gram-negative bacteria that is a widely used model organism in Biology, was used on all samples. *E. coli* strain BL21 (Thermo Fischer) was grown from glycerol stock (stored in −80 °C) by inoculating Lysogeny Broth (LB) at 37 °C for 24 h until growth is saturated in LB. After 24 h, the *E. coli* in LB was diluted to 1:2 and 1:10 aliquots in sterile LB. 10 µL of each diluted *E. coli* cultural were dispensed as a solution on the surfaces of our control and experimental surfaces. These surfaces with their aliquots of diluted *E. coli* were dried in a laminar flow hood for 15 min then incubated in a 37 °C incubator for 4 h increments. After the incubation time, the *E. coli* was harvested from each surface using a micropipette and 10 µL of LB and placed in a 1 mL test tube.

For determining the growth curves, aliquots of the harvested *E. coli* were placed in a 96-well optical density plate (Greiner Bio-One) for spectroscopy. In parallel, 300 µL of LB were placed into each well in triplicate to each harvested *E. coli* sample. The 96-well plate also had LB blanks present for calibration. The spectrophotometer (SpectraMax M2, Molecular Devices) was programmed to maintain a temperature of 37 °C for 20 h, to shake the plate for 5 s before a reading took place, to read each well at 600 nm wavelength, and to read every 30 min. After 20 h, the raw data was analyzed by averaging the triplicate data, and the growth curves of the *E. coli* were obtained.

The morphology of the self-assembled sphere seeds and the GLAD features grown on sphere seeds were determined by Scanning Electron Microscopy (SEM, Apreo). The hydrophobicity of the samples was determined by an optical tensiometer (Theta Lite).

## Results and discussion

**Figure 4:**
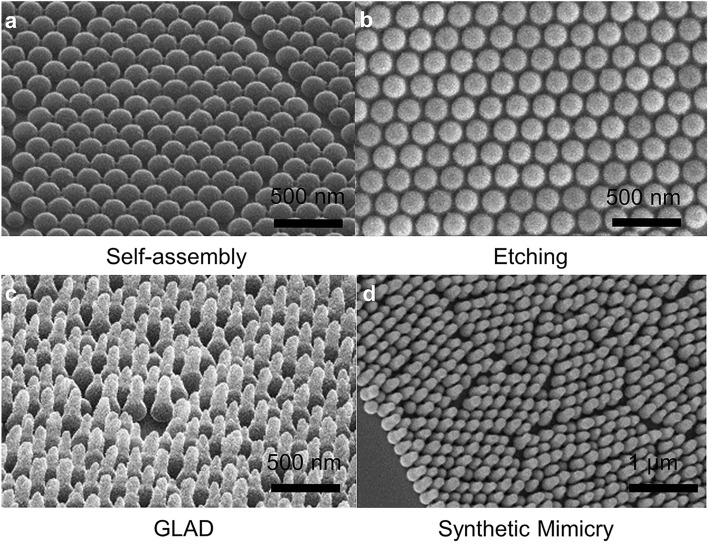
SEM images of the synthetic mimicry samples at various stages along the fabrication process. (**a**) After self-assembly of the nanospheres, (**b**) after plasma etching, (**c**) after GLAD deposition nanopillar cones, (**d**) after applying the parylene protection layer.

Figure 4 shows the SEM images of the synthetic mimicry samples at various stages along the fabrication process. Figure 4a shows hexagonally close packed nanospheres right after self-assembly. After 20 s of plasma etching, the nanospheres are separated and have a diameter of *D* ~ 150 nm. The nanospheres retain a spherical shape after etching, as shown in Fig. 4b. As designed, GLAD grows *d* = 90 nm nanopillars on top of the sphere seeds. The SEM micrograph of the synthetic mimicry sample shown in Fig. 4c (both quality and quantity) matches the actual cicada wing structures (as in Fig. 1b). The protection layer of parylene helps with the adhesion with the pillars to the sphere seeds. Figure 4d shows the nanopillar cones after application of the parylene layer; the diameter of the nanopillars increases to ~ 120 nm. The morphology of the nanopillar cone array is maintained after application of the protection layer.

**Figure 5:**
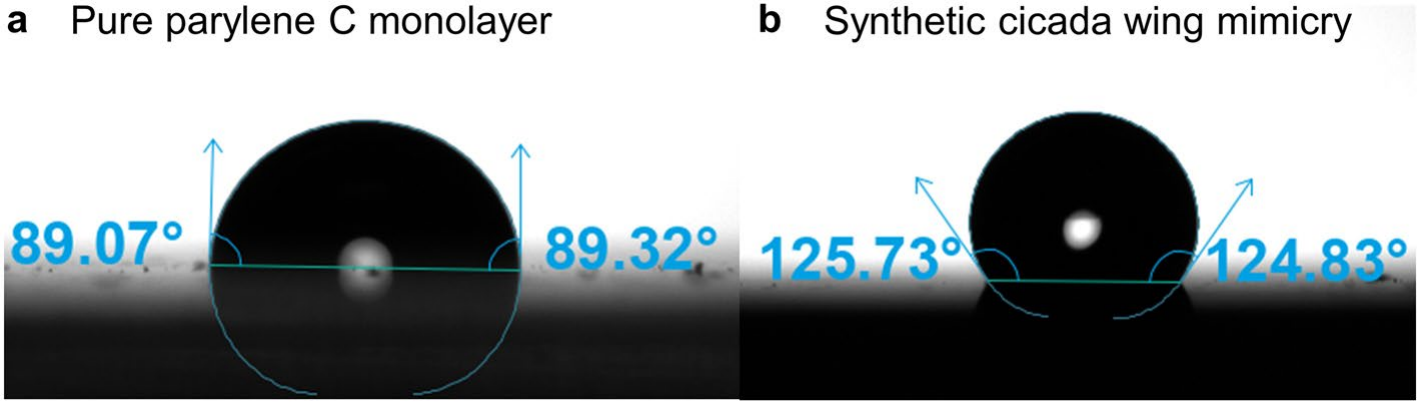
Water contact angle measurement on (**a**) pure parylene C monolayer, and (**b**) synthetic cicada wing mimicry.

The water contact angle was measured for the synthetic cicada wing mimicries in order to determine the hydrophobicity. Deionized (DI) water droplets (4 µL) were dispensed onto the samples. Due to poor adhesion of the germanium nanopillars on the nanosphere seeds, the nanopillars fell off the seeds after being immersed in water when no protection layer is added. With the protection layer, the nanostructures are robust in water, and the water contact angle could be obtained. Figure 5 shows one frame of a water droplet on the samples during the measurements. The average water contact angle is 125° on the synthetic mimicries, as shown in Fig. 5b, indicating that the synthetic mimicry is hydrophobic.

The hydrophobicity of the synthetic mimicries may be due to the nanostructure of the surface and the parylene protection layer. To further understand the mechanism of the hydrophobicity of the mimicry samples, the water contact angle test on bare parylene C monolayer (same thickness on the same substrate of planar glass slide) was conducted, and the water contact angle is only ~ 89˚, as shown in Fig. 5a. This means the parylene C is not a material with intrinsic low surface energy. Since the parylene protection layer is not hydrophobic, the hydrophobicity of the synthetic mimicries is only based on the nanostructures on the surfaces.

**Figure 6:**
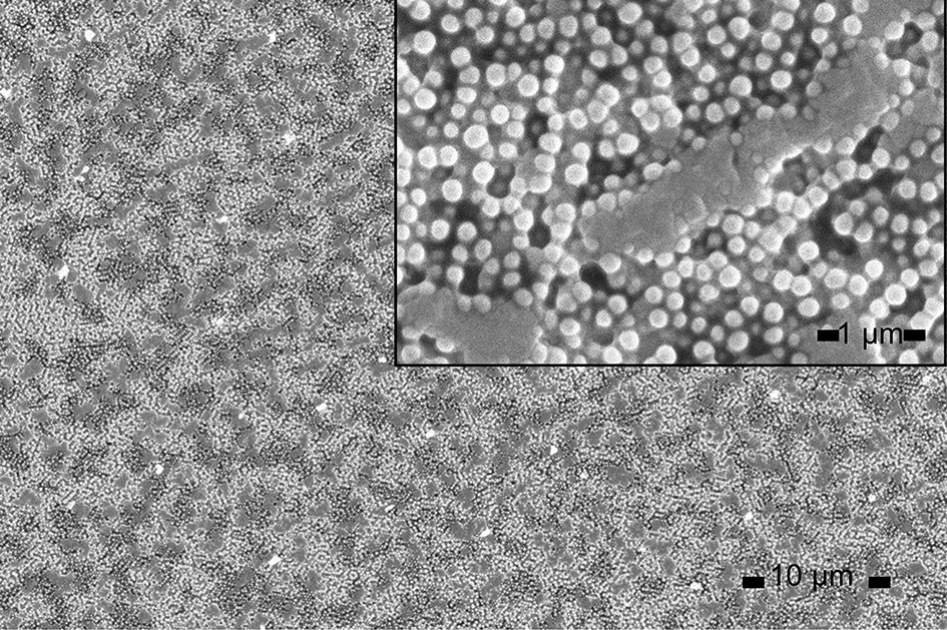
SEM images of the puncturing mechanism of the cicada wing mimicry.

**Figure 7:**
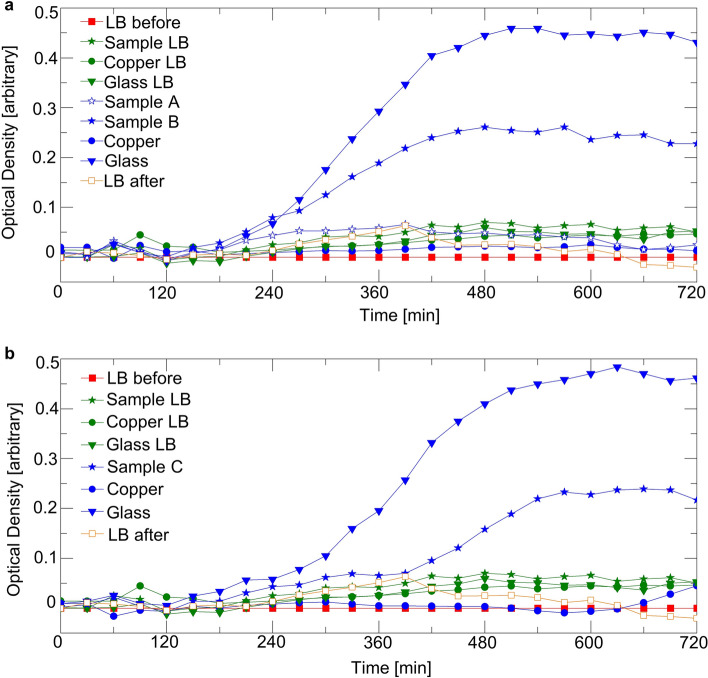
Spectrophotometer results for recording 12 h of *E. coli* growth on the cicada wing replica with dilution ratios of (**a**) 1:2 and (**b**) 1:10.

Figure 6 is an SEM image showing the killing mechanism of the synthetic cicada wing mimicry. Dead *E. coli* (darker lines) are spread all over the area of interest. The zoomed-in image shows that the nanopillar cones punctured the bacteria, with some of the pillars entirely going through the *E. coli*. The same bacteria-killing mechanism is validated by the growth curves of the *E. coli*, as shown in Fig. 7. As summarized in Table 1, the red and orange curves are the growth curves for the bacteria-free LB medium before and after the tests. The 0 optical density (OD) flat lines of the curves shows that the LB used was not contaminated. The green curves show the bacteria growth of all the samples and controls before the bacteria tests. The flat curves show that all the samples and controls are sterile before applying *E. coli*. The blue curves are the growth curves of the *E. coli* on samples (A–C) and controls (copper and glass slide).

**Table 1:** Explanations of the captions in Fig. 7.

Legend	Color, symbol	Explanation
LB before	Red, square	Test of broth before cultivating bacteria, to verify the broth is not contaminated
Sample/copper/glass LB	Green, star/circle/triangle	Test of sample/copper/glass surfaces by LB before cultivating bacteria, to verify surfaces are not contaminated
Sample A/B/C	Blue, star (open) in (a)/star (solid) in (a)/star (solid) in (b)	Test of samples A/B/C by LB with bacteria
Copper/glass	Blue, star/circle	Test of copper/glass by LB with bacteria, as positive and negative controls
LB after	Orange, square	Test of the original broth to verify the broth is not contaminated

Figure 7a shows experimental the growth curves when the dilution of the *E. coli* in LB is 1:2. The blue curve with circle symbols shows that the negative control of the copper had no *E. coli* growth. The blue curve with triangle symbols shows that the positive control of the glass had *E. coli* growth. The synthetic mimicry GLAD samples A and B are indicated by the blue curves with open and solid star symbols, respectively. GLAD Sample A showed no bacteria growth, while GLAD Sample B showed minor growth of the *E. coli*. Similarly, Fig. 7b shows the growth curves when the dilution of the *E. coli* in LB is 1:10. GLAD Sample C prohibited the growth of *E. coli* as shown in the blue curve with star symbols.

## Conclusion

In this paper, we introduced the reproduction of antimicrobial surfaces inspired by cicada wings. The synthesis of cicada wing nanostructures (nanopillar cones) is performed by GLAD and self-assembly: the sphere seeds are well designed for GLAD before being self-assembled on the substrate, while the GLAD parameters are adjusted and the pillars on top are successfully created to replicate the 3D nanofeature arrays on cicada wing surfaces. The reproduced nanofeatures possess the properties of cicada wings, including being hydrophobic and antimicrobial. The SEM images show the *E. coli* are punctured and killed by the surfaces, which is validated by the flat 12-h growth curve of *E. coli*. The large-area cicada-wing-mimicry antimicrobial surfaces are potentially useful in the food and biomedical industries^32,33^.

## Data Availability

The data in the current study available from the corresponding author on reasonable request.
